# Ventilation and Sanitation

**Published:** 1919-01

**Authors:** 


### Ventilation and Sanitation.

The assertion was made recently by W. N. Martin, a Y. M. C. A. educational secretary that while America is, as it should be, heartbroken over the 36,000 of our boys killed in this fight with the Germans, it is apparently little concerned that we killed in the same time 400,000 of our citizens in the United States by closing the doors and windows of our schools, churches, and homes and allowing them to die of that foul disease, tuberculosis. Every city, town and village throughout the land should join with the physicians in a systematic effort to show that much of the disease of the country can be prevented by proper sanitation and ventilation. Under some conditions illness is criminal.

